# Microstructural Evolution and Refinement Mechanism of a Beta–Gamma TiAl-Based Alloy during Multidirectional Isothermal Forging

**DOI:** 10.3390/ma12152496

**Published:** 2019-08-06

**Authors:** Kai Zhu, Shoujiang Qu, Aihan Feng, Jingli Sun, Jun Shen

**Affiliations:** 1 School of Materials Science and Engineering, Tongji University, Shanghai 201804, China; 2 Shanghai Key Laboratory of Development and Application for Metal-functional Materials, Tongji University, Shanghai 201804, China; 3 Shanghai Spaceflight Precision Machinery Institute, Shanghai 201600, China; 4 College of Mechatronics and Control Engineering, Shenzhen University, Shenzhen 518060, China

**Keywords:** TiAl-based alloy, microstructure characterization, refinement mechanism, multidirectional isothermal forging (MDIF)

## Abstract

Multidirectional isothermal forging (MDIF) was used on a Ti-44Al-4Nb-1.5Cr-0.5Mo-0.2B (at. %) alloy to obtain a crack-free pancake. The microstructural evolution, such as dynamic recovery and recrystallization behavior, were investigated using electron backscattered diffraction and transmission electron microscopy methods. The MDIF broke down the initial near-lamellar microstructure and produced a refined and homogeneous duplex microstructure. γ grains were effectively refined from 3.6 μm to 1.6 μm after the second step of isothermal forging. The ultimate tensile strength at ambient temperature and the elongation at 800 °C increased significantly after isothermal forging. β/B2→α_2_ transition occurred during intermediate annealing, and α_2_ + γ→β/B2 transition occurred during the second step of isothermal forging. The refinement mechanism of the first-step isothermal forging process involved the conversion of the lamellar structure and discontinuous dynamic recrystallization (DDRX) of γ grains in the original mixture-phase region. The lamellar conversion included continuous dynamic recrystallization and DDRX of the γ laths and bugling of the γ phase. DDRX behavior of γ grains dominated the refinement mechanism of the second step of isothermal forging.

## 1. Introduction

γ-TiAl-based alloys are considered highly promising materials for aeroengine and automotive applications because of their attractive properties, including low density, high-temperature strength, good oxidation, and creep resistance at elevated temperatures [1,2,3,4]. Recently, an advanced γ-TiAl-based alloy called beta–gamma TiAl-based alloy has gradually aroused researchers’ interest because it has good hot workability, homogeneous microstructures, weak textures, and minimal segregation [5]. Different from traditional γ-TiAl-based alloys, which contain two basic phases (i.e., γ-TiAl and α_2_-Ti_3_Al) and often small amounts of the β_0_ phase, the beta–gamma TiAl-based alloy is a multiphase alloy that consists of three major phases (i.e., γ-TiAl, α_2_-Ti_3_Al and β_0_-Ti) and an additional phase (i.e., ω_0_-Ti_4_Al_3_Nb), which usually co-exists with the β_0_ phase [6,7]. The β phase has a body centered cubic (bcc) structure and can work as a lubricant during hot processing, thereby facilitating the plastic flow and improving the formability of γ-TiAl-based alloys at elevated temperatures [8]. Therefore, the beta–gamma TiAl-based alloy is suitable for hot processing without cracks, and can produce a fine-grained (FG) microstructure with improved mechanical properties [9,10,11]. The typical hot processing for beta–gamma TiAl-based alloys, such as isothermal forging, hot extrusion, and rolling, is also called thermomechanical treatment [12,13,14].

Isothermal forging is an economical and developed forging method used for manufacturing critical components, especially those that are made of difficult-to-process materials [15]. Isothermal forging equipment can provide a relatively isothermal environment and a slow strain rate. Changing the isothermal forging direction is a developed forging technology called multidirectional isothermal forging (MDIF). This method can help deduce the deformation dead zone and refine the microstructure. Therefore, MDIF is an effective approach for producing large billets with FG microstructures [16]. Zherebtsov et al. [17] produced a homogeneous sub-microcrystalline structure in Ti-6Al-4V billet with improved mechanical properties by MDIF. Salishchev et al. [18,19] applied MDIF to γ-TiAl-based alloys to produce a homogeneous sub-microcrystalline structure and achieved low-temperature superplasticity. In this study, MDIF was applied in a beta–gamma TiAl-based alloy to breakdown as-cast microstructure and produce a bulk FG pancake.

Previous studies have mainly focused on hot-working behavior, including deformation mechanisms, phase transformation, and dynamic recrystallization (DRX) behavior of various γ-TiAl-based alloys (e.g., traditional γ-TiAl-based alloys, high Nb-containing TiAl-based alloys, and β-solidifying TiAl-based alloys) [20,21,22,23]. However, beta–gamma TiAl-based alloys are unique and complex due to the addition of multiple β-stabilized elements. Phase transitions accompanied with DRX occur during the hot deformation of beta–gamma TiAl-based alloys, and many details about this phenomenon are not yet fully understood [24]. Moreover, MDIF is a special type of forging that involves multistep forging and intermediate heat treatment. Studies on TiAl-based alloys, even beta–gamma TiAl-based alloys, produced by MDIF are few. This study combined MDIF with advanced beta–gamma TiAl-based alloys, with the expectation that we could produce a larger size billet with FG structure and improved mechanical properties. The FG structure is beneficial for secondary processes, such as wrought processing, rolling [1], and has the potential to obtain low-temperature superplasticity [25].Furthermore, details about the phase transformation and the conversion of the microstructure of a beta–gamma TiAl-based alloy from a lamellar structure into a duplex structure during MDIF are ambiguous.

In the present study, the phase transformation, DRX behavior, and grain boundary (GB) characteristics were investigated in a beta–gamma TiAl-based alloy. The study aimed to achieve a comprehensive understanding of the microstructural evolution and refinement mechanism of this alloy during MDIF.

## 2. Materials and Methods

In this work, the nominal composition of the beta–gamma TiAl-based alloy was Ti-44Al-4Nb-1.5Cr-0.5Mo-0.2B (at. %) (hereafter referred to as TiAlNbCrMo alloy). The ingot was prepared by double vacuum consumable arc melting technique, with dimensions of approximately φ197 mm × 285 mm. Subsequently, the as-cast ingot was subjected to hot isostatic pressing (HIP) at 1280 °C and P = 180 MPa for 1 h, and then 1300 °C and P = 180 MPa for 2 h, with subsequent furnace cooling under argon (purity: 99.999%) atmosphere. The sample for MDIF was cut from the HIP ingot with dimensions of φ60 mm × 100 mm.

**Figure 1:**
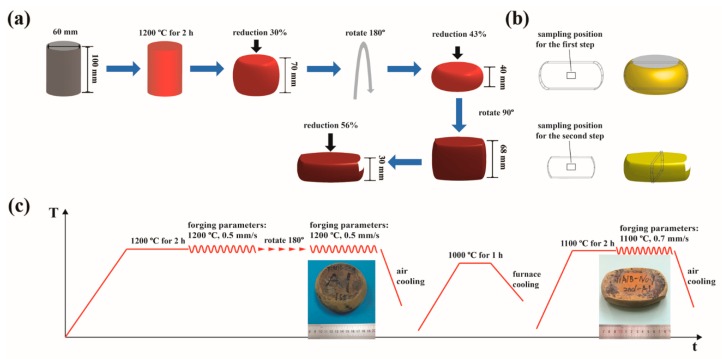
(**a**) Schematic of MDIF (multidirectional isothermal forging); (**b**) sampling position for the first and second steps of isothermal forging; (**c**) parameters of MDIF and intermediate annealing, appearance of the first-step and the second-step isothermally forged pancakes.

Figure 1a shows the entire MDIF process. Rotating 180° of the two-step isothermal forging in the one direction is to offset the uneven deformation caused by the temperature difference between the top and bottom of the furnace. In this study, this two-step isothermal forging in the one direction was defined as the first step of isothermal forging, and the isothermal forging after 90° rotation was defined as the second step of isothermal forging. Prior to the 90° rotation, the isothermal forging pancake was canted by machining the curved faces and annealed at 1000 °C for 1 h to eliminate internal stress. To refine the microstructure, the isothermal forging temperature in the next stage decreased from 1200 °C to 1100 °C. The specimens for microstructural tests were cut from the mid-plane of the forged billet (Figure 1b). Figure 1c illustrates the parameters of MDIF and intermediate annealing. Figure 1c also displays the first-step and second-step isothermally forged pancakes of TiAlNbCrMo alloy. Sound surfaces and no cracks were visible in these two pancakes. Water-quenched (WQ) treatment was conducted at 1200 °C for 2 h on the small sample to obtain a high-temperature phase distribution. A small sample with dimensions of 8 mm × 8 mm × 3 mm was cut from the HIP ingot.

The microstructures of the HIP, heat-treated, and as-forged samples were investigated via scanning electron microscopy (SEM; Quanta 400 FEG, FEI Company, Hillsboro, OR, USA) in the backscattered electron mode at an acceleration voltage of 20 kV. The phase compositions, crystal orientation, and other related microstructural features were characterized using electron backscattered diffraction (EBSD; Oxford Instruments, Oxford, UK) with step sizes of 0.1–0.15 μm and a scanning electron microscope (Quanta 450, FEI Company, Hillsboro, OR, USA). The EBSD data were analyzed using HKL Channel 5 software (version 5.11.10405.0) (Oxford Instruments, Oxford, UK). The samples for SEM and EBSD characterization were electrochemically polished with a 6 vol. % perchloric acid, 34 vol. % n-butyl alcohol, and 60 vol. % methanol electrolytes that operated at 60 V and −30 °C. Transmission electron microscopy (TEM) characterization was conducted on a JEM-2100F (JEOL Ltd., Tokyo, Japan) at an acceleration voltage of 200 kV. Thin foils for TEM were prepared to a thickness of 60–80 μm through mechanical polishing and twin-jet electropolishing with a 6 vol. % perchloric acid, 34 vol. % n-butyl alcohol, and 60 vol. % methanol electrolytes that operated at 45 V and −30 °C.

## 3. Results

### 3.1. Microstructure Prior to Forging

**Figure 2:**
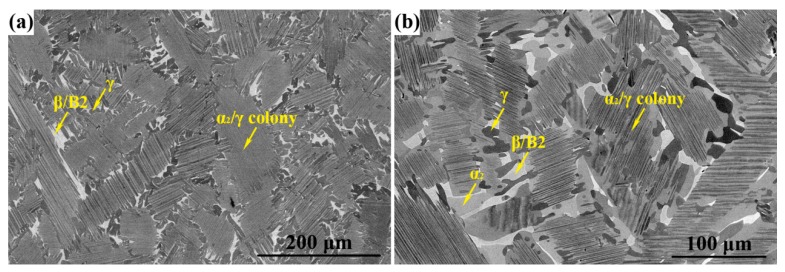
SEM (scanning electron microscopy) images of TiAlNbCrMo alloy: (**a**) after hot isostatic pressing (HIP); (**b**) 1200 °C/2 h/WQ. The γ phase appears as dark gray. The light gray contrast indicates α_2_/γ colonies, whereas the white ones correspond to the β/B2 phase.

The microstructure of HIP TiAlNbCrMo alloy was a so-called near-lamellar structure, which is divided into two parts, namely, α_2_/γ lamellar colonies and γ + B2 two-phase mixtures, (Figure 2a). α_2_/γ lamellar colonies were the predominant structure and their average size was ~100 μm. γ + B2 two-phase mixtures were located along colony boundaries. The B2 phase was a thermodynamically stable β phase below ordering temperature. Further details about the as-cast microstructure of TiAlNbCrMo alloy were investigated in a previous study [26]. Figure 2b displays the WQ microstructure of this alloy after maintaining at 1200 °C for 2 h. In this way, the actual microstructure prior to isothermal forging could be deduced. The image indicates that the size of α_2_/γ lamellar colonies reduced, and the mixture-phase (γ + α + β/B2) region increased. Previous research has shown that 1200 °C is within γ + α + β/B2 phase field of this alloy. When heating to 1200 °C, β/B2→α and γ + α_2_→α transitions occurred [27].

### 3.2. Microstructure Characterization during MDIF

**Figure 3:**
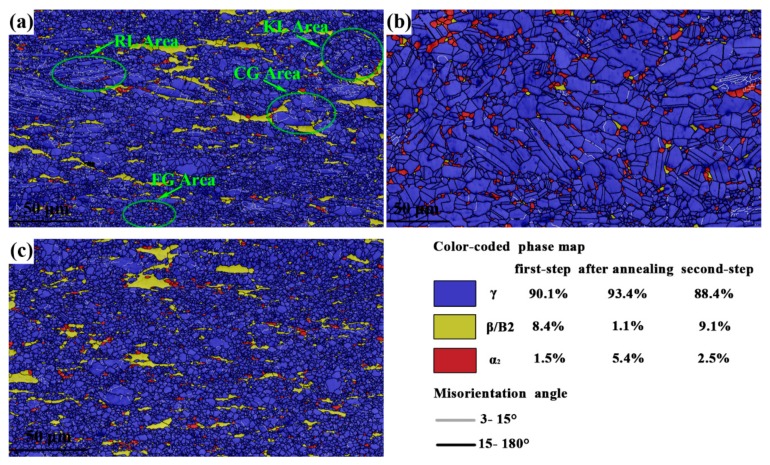
Phase distribution maps overlapped by GBs of TiAlNbCrMo alloy during MDIF: (**a**) first step of isothermal forging; (**b**) intermediate annealing at 1100 °C for 1 h; (**c**) second step of isothermal forging. The isothermal forging direction in all images is vertical. RL, KL, FG, and CG denote remnant lamellar, kinked lamellar, fine grain, and coarse grain, respectively.

**Figure 4:**
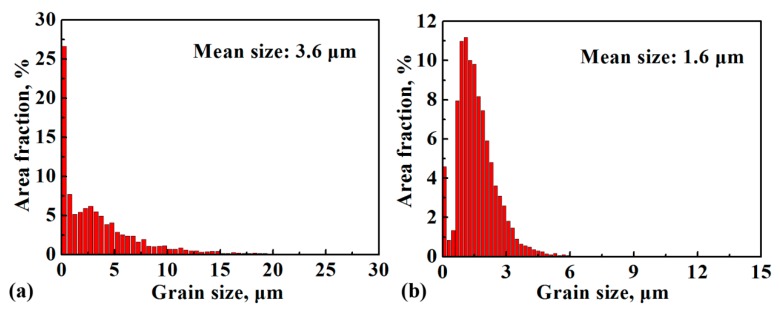
Distribution of the γ grain size of TiAlNbCrMo alloy: (**a**) after annealing at 1100 °C for 1 h; (**b**) after the second step of isothermal forging.

Figure 3 presents the phase distribution and GB maps of the first step of forging, intermediate annealing, and the second step of isothermal forging. The first-step isothermally forged microstructure consisted of approximately 90.1% γ, 1.5% α_2_, and 8.4% β/B2 phase. After the first step of forging, many lamellar colonies were broken into a FG duplex microstructure, denoted as the FG area in Figure 3a. In some areas, coarse γ grains containing lots of low-angle GBs (LAGBs, 3–15°) adjacent with flat β/B2 grains and trace α_2_ phase can be observed, denoted as the coarse grain (CG) area in Figure 3a. However, some remnant lamellar structures still existed in the first-step isothermally forged microstructure, denoted as the remnant lamellar (RL) area in Figure 4a. Kinked lamellar structures are also highlighted in Figure 3a (KL area) as a distinctive RL structure, rather than other, bent perpendicular to the forging direction lamellar structures. The intermediate annealing microstructure consisted of approximately 93.4% γ, 5.4% α_2_, and 1.1% β/B2 phase, and it contained more than 95% high-angle GBs (HAGBs, 15–180°, Figure 3b), which were only 81% in the first-step isothermally forged microstructure. In comparison with the first-step isothermally forged microstructure, the content of the γ phase was nearly consistent with that in the intermediate annealing microstructure. Meanwhile, the content of the α_2_ phase increased, the B2 phase decreased, and the amount of HAGBs increased. This result indicates that β/B2→α_2_ transition occurred, and static recrystallization took place during intermediate annealing at 1100 °C. Furthermore, the intermediate annealing treatment was beneficial to reduce the RL structure and produced an equiaxed duplex microstructure (Figure 3b). Niu et al. [24] reported that post-forging annealing within γ + α_2_ + β/B2 for a beta–gamma TiAl-based alloy could entirely remove the RL structures. The microstructure of the second-step isothermal forging was characterized by approximately 88.4% γ, 2.5% α_2_, and 9.1% β/B2 phase. After the second step of isothermal forging, the relatively coarse duplex microstructure was transformed into a FG duplex microstructure (Figure 3c). In comparison with the intermediate annealing microstructure, the content of the γ and α_2_ phases slightly decreased, and the B2 phase increased. Therefore, α_2_ + γ→β/B2 transition occurred during the second step of isothermal forging. Figure 4 shows the size distribution of γ grain and the value of its statistical mean size of the intermediate annealing microstructure and the second-step isothermally forged microstructure. The mean size of γ grains could be accounted after the first step of forging due to the existence of RL structures. However, the result clearly demonstrates that the isothermal forging effectively refined the grains from 3.6 μm to 1.6 μm.

**Figure 5:**
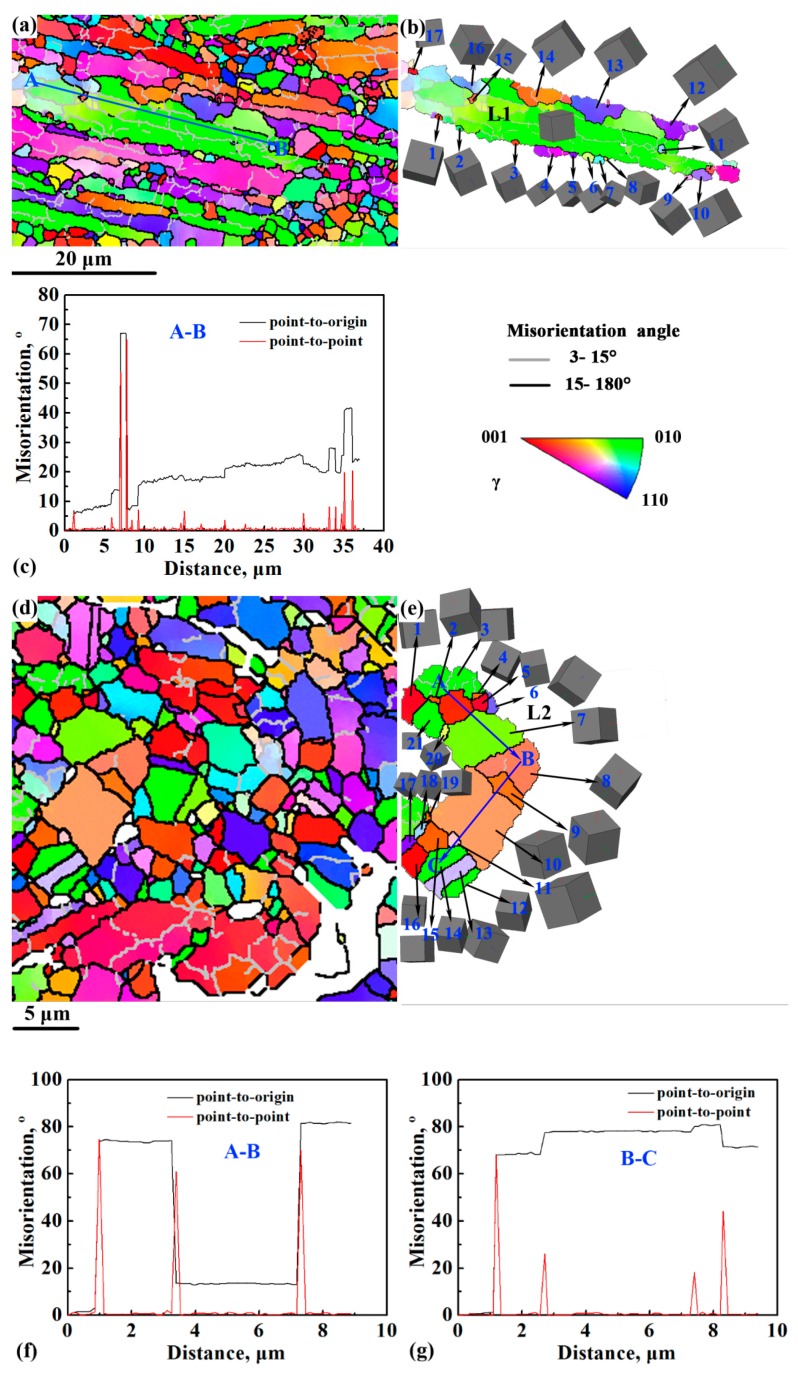
RL area in the first-step isothermally forged microstructure of TiAlNbCrMo alloy: (**a**) inverse pole figure (IPF) map of γ grains; (**b**) IPF map and corresponding crystallographic orientations of L1 selected in (**a**); (**c**) line profiles of misorientation angles along arrow AB in (**b**). KL area in the first-step isothermally forged microstructure: (**d**) IPF map of γ grains; (**e**) IPF map and corresponding crystallographic orientations of L2 selected in (**d**); (**f**,**g**) line profiles of misorientation angle along the arrows AB and BC in (**e**).

Figure 5 shows the EBSD results of the RL and KL areas in the first-step isothermally forged microstructure. The γ phase was selected as the research object because it was the dominant phase in these areas, and α_2_ laths were extremely thin and difficult to detect by EBSD. Figure 7 presents the details of these areas. Figure 5a indicates that RL area consisted of some remnant γ lamellae and DRXed grains along the lamellar interfaces. In addition, remnant γ lamellae contained many LAGBs due to accumulated dislocations or the existence of sub-GBs [28]. Notably, the remnant γ lamellae were nearly perpendicular to the isothermal forging direction. Figure 5b shows a typical remnant γ lamella called L1. Figure 5b shows the grains adjacent and within L1, labeled 1–17. Among them, 13 FGs (1–11, 15 and 17) and 4 large grains (12, 13, 14 and 16) can be observed. Grains 11 and 15 formed inside L1, whereas the other grains (1–10, 12–14, 16, and 17) formed at the serrated lamellar boundaries, which is the typical feature of discontinuous DRX (DDRX) [29]. The line profile of point-to-origin along the blue arrow AB (Figure 5c) indicates that the misorientation angle gradually increased up to 40°. Thus, the continuous change of crystal orientation occurred in L1. The point-to-point line profile (Figure 5c) indicates that two HAGBs formed at a distance of 7 μm and 35 μm, which corresponded to the formation of grains 11 and 15. The formation of grains 11 and 15 was associated with the absorption of dislocations or sub-GBs within L1. These features can be classified as a continuous DRX (CDRX) mechanism [29]. Figure 5d indicates that the KL area consisted of kinked γ lamellae and DRXed grains. Some γ laths were broken down into FGs, and many HAGBs were found within the original γ laths. In comparison with the RL area, the LAGBs still existed in the γ laths. However, their amount decreased remarkably, because the kinking angle in this area was over 60°, thereby resulting in extremely high local strain, which provides the driving pressure for phase transformation and recrystallization [30]. Figure 5e exhibits a selected kinked γ lamella and the grains adjacent and within L2, labeled 1–20. Fine DRXed grains 2, 4, 11, 17–20 were located within L2. The point-to-point line profiles along the blue arrows AB (Figure 5e) and BC (Figure 5f) indicate that the misorientation angle slightly changed within a single grain. This phenomenon implies that the origin lath was broken down into several grains due to DRX.

**Figure 6:**
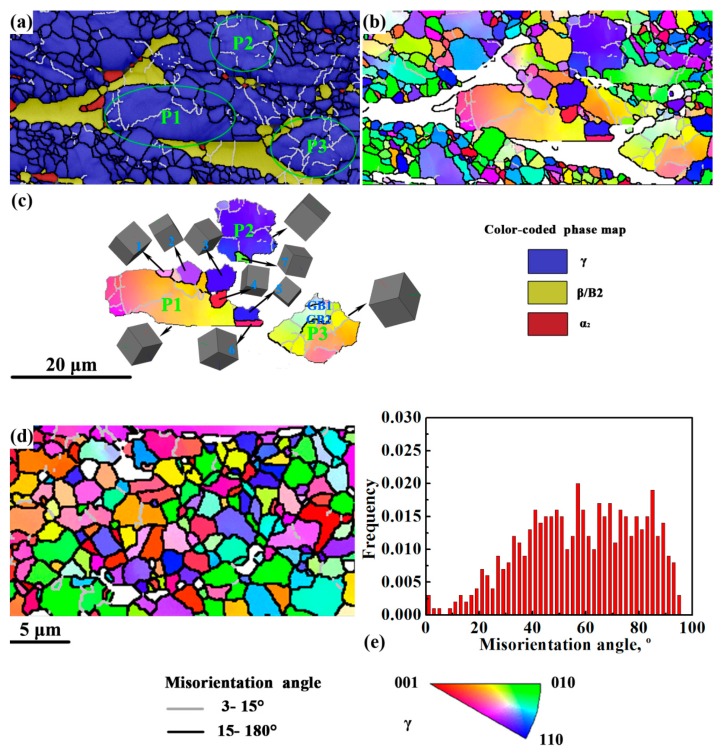
CG area in the first-step isothermally forged microstructure of TiAlNbCrMo alloy: (**a**) phase map; (**b**) IPF map of γ grains; (**c**) IPF map and corresponding crystallographic orientations of P1, P2, and P3 selected in (**b**). FG area in the first-step isothermally forged microstructure: (**d**) IPF map of γ grains; (**e**) distribution of GB misorientations.

Figure 6 shows the EBSD results of the CG and FG areas in the first-step isothermally forged microstructure. In the CG area, the γ phase remained the major phase, accompanied by coarse and flat β/B2 grains and trace α_2_ phase (Figure 6a). Few LAGBs existed in the β/B2 grains; thus, the β/B2 phase was prone to dynamic recovery (DRV) during hot deformation, due to its high stacking fault energy (SFE) [31]. Figure 6b shows the IPF map of γ grains in the CG area. The results show that the CG area consisted of several unDRXed γ grains and numerous fine DRXed γ grains. Three unDRXed (parent) grains (i.e., P1, P2, and P3) and some adjacent grains, labeled 1–7, were selected for investigation. Grains 1–7 were located at the serrated GBs in P1 and P2. Meanwhile, the GBs of grains 1–4 and 7 were observed to bulge toward the parent grains. The aforementioned observations indicate that the DDRX dominated the DRX behavior in coarse γ grains during isothermal forging. In addition, two HAGBs, labeled GB1 and GB2, were observed in grain P3. The misorientation angles of GB1 and GB2 were 28° and 22°, respectively. Meanwhile, the misorientation angles of the sub-GBs adjacent to them were between 5–10°. The accumulation of sub-GBs may lead to the formation of GBs in unDRXed γ grains, which is the feature of CDRX mechanism. In the FG area, fine and equiaxed γ grains were observed (Figure 6d). Figure 6e shows that the orientation of γ grains presented random distribution on a high misorientation angle. These results show that γ grains completely recrystallized in the FG area.

**Figure 7:**
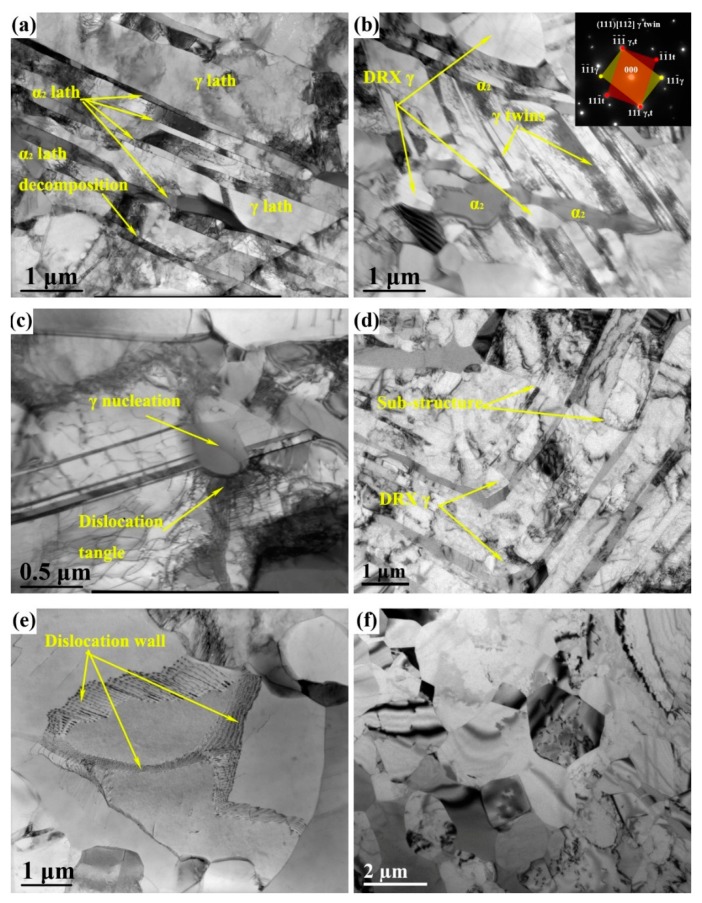
TEM (transmission electron microscopy) images of the first-step isothermally forged microstructure of TiAlNbCrMo alloy: (**a**) slightly deformed remnant lamellae; (**b**) remnant lamellae with γ twins and DRX γ grains. Selected area electron diffraction (SAED) pattern in the corner corresponding to the γ twins; (**c**) γ nucleation and dislocation tangle; (**d**) kinked lamellae; (**e**) dislocation wall within a γ grain; (**f**) refined grains.

Figure 7 shows the typical microstructures of different areas of the TiAlNbCrMo alloy after the first step of isothermal forging. Figure 7a presents the remnant lamellae composed of many thin (<100 nm) α_2_ laths, accompanied by deformed γ laths. The dislocation density in the γ laths was higher than that in the α_2_ laths; thus, the coarsened γ laths bore the main strain, whereas the DRV in α_2_, which lathed during hot deformation, consumed the dislocations, which was consistent with a previous study [32]. In addition, the decomposition of α_2_ laths was also observed in Figure 7a, indicating that α_2_→γ transition also occurred. This similar phenomenon was also found in a previous study [33]. Figure 7b shows a heavily deformed RL area. In this area, the perfect Blackburn orientation relationship [34] between the α_2_ and γ lath was destroyed. High-density dislocations and deformation twins (proven by the SAED pattern) were found in γ laths. Moreover, several DRXed grains were observed at the lamellar interfaces with the feature of GB bugling. During deformation, dislocations were prone to piling up at the twin boundary and lamellar interfaces, which provided the driving force of recrystallization. Dislocations that piled up at the twin boundaries could transfer into sub-GBs, and dislocations that tangled at the lamellar interfaces could trigger the DDRX process of γ grains with further deformation. Hao et al. [21] supported this conjecture. Figure 7c shows newly formed DRXed γ grains at the dislocated tangled position along the lamellar interface, thereby confirming the previous conjecture. The growth of DRXed γ grains into α_2_ laths by bugling is a phase boundary bugling mechanism, which is the breakdown mechanism of α_2_ laths [28]. Figure 7d shows a KL structure. Several DRXed γ grains were found in the structure with the highest local strain, and some sub-GBs were observed in γ laths in Figure 7d. The lamellae were reoriented with respect to the forging direction due to kinking, which may have induced high local strain and shearing stress among the lamellar structure. This finding suggests that the high local strain provided the driving thermodynamic force for the observed recrystallization, and the shearing stress led to the formation of sub-GBs. These sub-GBs may have rotated into HAGBs by sliding along the GBs during hot deformation, combined with the observed results in Figure 5b,e. Figure 7e shows the dislocation walls in an unDRXed γ grain. This phenomenon was caused by the sub-GBs found in Figure 6c. Figure 7f shows the duplex structure with fine recrystallized γ grains and α_2_ grains nearly free of dislocations by DRV of the α/α2 and β/B2 phases, phase boundary bugling, and DRX of γ grains.

**Figure 8:**
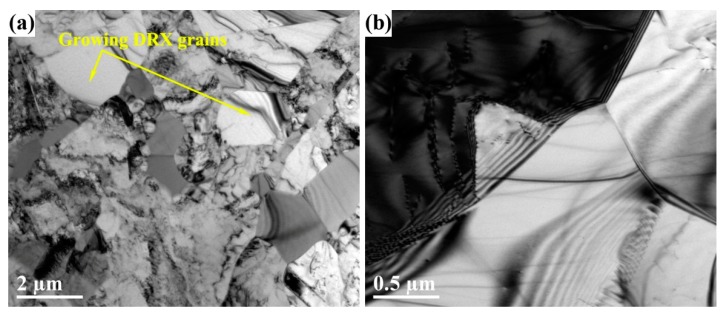
TEM images of the second-step isothermally forged microstructure of TiAlNbCrMo alloy: (**a**) growing DRX γ grains; (**b**) GB features of γ grains.

Figure 8 shows the microstructures of different areas of TiAlNbCrMo alloy after the second step of isothermal forging. Figure 8a displays several γ grains free of dislocations because DRX underwent the second step of isothermal forging. Figure 8b illustrates the manner in which the DRXed γ grains nucleated and grew due to GB bugling. Before the second step of isothermal forging, the microstructure of TiAlNbCrMo alloy was predominantly equiaxed γ grains, with a small amount of the α_2_ and β/B2 phases (Figure 3b). After the second step of isothermal forging, the size of the γ grains was refined due to DRX. 

## 4. Discussion

### 4.1. Advantage of MDIF

The results in Section 3.1 show that the microstructure prior to isothermal forging was divided into two parts, namely, the α_2_/γ lamellar colonies and γ + α+ β/B2 mixture phases. Previous studies have shown that α_2_/γ lamellar colonies present a strong anisotropic plastic behavior [35,36,37,38]. The strength of the lamellar colonies is remarkably affected by the angle φ between the lamellar orientation and the loading axis. When the angle φ is close to 45°, the strength is the lowest, and when the angle φ is 0° or 90°, the strength increases [38]. Consequently, the recrystallization behavior of the lamellar colony exhibits anisotropy [36]. A previous work [26] indicated that the colony orientation transfers into an orientation perpendicular to or close to perpendicular to the compression direction by kinking and rotation during deformation. The mixture phases along the colony boundaries have different hot workabilities at high temperatures. However, their workabilities are better than the lamellar colony, and play a role of coordinating strain among various orientation colonies [39,40]. α and β phases are prone to DRV, whereas the γ phase is prone to DRX during hot deformation, due to their different SFEs [1].This is the cause of CG area that can be found in Figure 3. Therefore, via the first step of isothermal forging, partial original colonies were broken down into FGs (FG area), some colonies remained (RL area), and the mixture-phase area converted into CG area. It is difficult to reduce the RL area by just a one-step isothermal forging process [24,28]. The intermediate annealing treatment helps to eliminate the RL structures because the static recrystallization of γ grains, and is an effective route for the decomposition of lamellar structures [41]. After the first step of isothermal forging, the RL transferred into an orientation that was nearly perpendicular to the forging direction (Figure 3a). The original RL turned into an orientation that was nearly parallel to the forging direction, due to the 90° rotation before the second step of isothermal forging. Therefore, the second step of isothermal forging effectively broke down the lamellar structure and produced a refined and homogeneous microstructure. A similar method has been used in Ti-6Al-4V alloy to produce large-scale billets with a homogeneous structure [16,17]. The mechanical properties of TiAlNbCrMo alloy in different states are listed in Table 1. The tensile strength at ambient temperature was improved significantly after isothermal forging, due to the fine and homogenous duplex microstructure. The ultimate tensile strength at ambient temperature increased from about 536 MPa to 771 MPa after the first step of isothermal forging, but decreased slightly to 735 MPa after the second step of isothermal forging. The ultimate tensile strength at 800 °C decreased from about 552 MPa to 515 MPa after the first step of isothermal forging, and to 486 MPa after the second step of isothermal forging. The elongation at ambient temperature decreased from 0.39% to 0.1% after isothermal forging, but the elongation at 800 °C increased significantly.

### 4.2. Refinement Mechanism during MDIF

Obviously, the microstructure of TiAlNbCrMo alloy was refined from a near lamellar structure with coarse colonies into a duplex structure by the first step of isothermal forging, and from a relatively CG duplex microstructure into a FG duplex microstructure by the second step of isothermal forging. However, the refinement mechanism of the first step of isothermal forging was different from that of the second step. After the first step of isothermal forging, the microstructure converted from a near-lamellar structure into a mixed structure with RL, KL, CG and FG areas. Two transformation mechanisms were observed during that process. One was the conversion of the lamellar structure, and the other was the refinement of γ grains in the original mixture-phase region. The lamellar colony was actually a lamella package configuration with a γ lamella matrix and a certain amount of α_2_ lamellae. This type of structure exhibits an “instability” deformation mode under compressive deformation [42]. In addition, kink band and shear localizations were present in the instability deformation mode, which led to local strain concentration. The plastic local strain in the lamellar structure led to formation dislocation pile-ups or deformation twins impeded by lamellar boundaries; moreover, the deformation structures were remarkably affected by the orientation with respect to the loading axis [43,44]. The high-density dislocations and deformation twins provided the driving force for the subsequent recovery or recrystallization (Figure 7). Meanwhile, the DRX mechanism in the lamellar structure can be ascribed to the combination of DDRX and CDRX mechanisms in the γ laths, as proven by the features shown in Figure 5 and Figure 7. In addition, the spheroidization of the α_2_ laths resulted from the bugling of the γ phase (Figure 7b). The refinement mechanism of the γ grains in the original mixture-phase region can be deduced from the features in the CG area (Figure 6a–c). The results show that the DDRX mechanism dominated the DRX behavior. Some strain-induced HAGBs were observed in the CG area; however, no CDRX γ grains were detected. Likewise, after the second step of isothermal forging, the conversion of the microstructure referred to the refinement of γ grains due to DRX. The nucleation of these DRXed γ grains, which is a typical feature of the DDRX mechanism, occurred at pre-existing HAGBs via the local strain-induced GB migration (Figure 8b) [29]. Therefore, DDRX dominated the refinement mechanism during the second step of isothermal forging. DRX behavior is affected by many factors, such as SFE, thermomechanical processing conditions, the initial grain sizes, chemistry and microchemistry of the materials, the second phase particles, etc. [45]. The γ phase was found to have a low SFE, especially with the ternary additions (e.g., Nb, Mo, and Cr) [22,46]. In many studies, the DDRX mechanism has been found to dominate the DRX behavior in γ-TiAl-based alloys during hot deformation [33,47,48]. However, some studies suggest that the CDRX mechanism could dominate the DRX behavior at higher temperatures or lower strain rates [12,49], which just corresponds to the deformation condition of MIDF found in the present study. This may help to explain the CDRX mechanism found in the γ laths and the formation of GB1 and GB2 in P3 (Figure 6c).

## 5. Conclusions

In this study, MDIF was applied to a beta–gamma TiAl-based alloy to achieve a fine and homogeneous microstructure on a large scale and with good mechanical properties. The recovery, recrystallization behavior, and microstructure evolution were systematically investigated using EBSD and TEM characterization. The main conclusions are summarized as follows:

(1) A crack-free pancake with a sound surface of TiAlNbCrMo alloy was produced by MDIF. The MDIF broke down the initial near-lamellar microstructure (i.e., α_2_/γ lamellar colonies and γ + B2 two-phase mixtures) and produced a refined and homogeneous duplex microstructure (i.e., γ and B2 phase).
(2) The β/B2→α_2_ transition occurred during intermediate annealing, and the α_2_ + γ→β/B2 transition occurred during the second step of isothermal forging, after which the γ grains were effectively refined from 3.6 μm to 1.6 μm.
(3) The refinement mechanism of the first step of isothermal forging process involved the conversion of the lamellar structure and DDRX of γ grains in the original mixture-phase region. The conversion of the lamellar included CDRX and DDRX of the γ laths and the spheroidization of α_2_ laths by bugling of the γ phase. The refinement mechanism of the second step of isothermal forging was mainly the DDRX behavior of the γ grains.
(4) The ultimate tensile strength at ambient temperature and the elongation at 800 °C increased significantly after isothermal forging, but the elongation at ambient temperature decreased.

## Figures and Tables

**Table 1:** Tensile properties of TiAlNbCrMo alloy in different states.

State	Test Temperature	Yield Strength (MPa)	Ultimate Tensile Strength (MPa)	Elongation (%)
HIP	Room temperature	497	536	0.39
	800 °C	444	552	8.22
After the first step of isothermal forging	Room temperature	-	771	0.1
	800 °C	470	515	91.9
After the second step of isothermal forging	Room temperature	-	735	0.1
	800 °C	421	486	75.2

## References

[1] Appel F., Paul J.D.H., Oehring M. (2011). Gamma Titanium Aluminide Alloys: Science and Technology.

[2] Kim Y.-W. (1995). Gamma titanium aluminides. JOM.

[3] Wu X.H. (2006). Review of alloy and process development of TiAl alloys. Intermetallics.

[4] Qu S.J., Tang S.Q., Feng A.H., Feng C., Shen J., Chen D.L. (2018). Microstructural evolution and high-temperature oxidation mechanisms of a titanium aluminide based alloy. Acta Mater..

[5] Kim Y.W., Kim S.L., Dimiduk D., Woodward C. (2008). Development of Beta Gamma Alloys: Opening Robust Processing and Greater Application Potential for TiAl-Base Alloys.

[6] Schloffer M., Rashkova B., Schöberl T., Schwaighofer E., Zhang Z., Clemens H., Mayer S. (2014). Evolution of the ω_o_ phase in a β-stabilized multi-phase TiAl alloy and its effect on hardness. Acta Mater..

[7] Huang Z.W. (2008). Ordered ω phases in a 4Zr-4Nb-containing TiAl-based alloy. Acta Mater..

[8] Wallgram W., Schmölzer T., Cha L., Das G., Güther V., Clemens H. (2009). Technology and mechanical properties of advanced γ-TiAl based alloys. Int. J. Mater. Res..

[9] Tetsui T. (2002). A newly developed hot worked TiAl alloy for blades and structural components. Scripta Mater..

[10] Tetsui T., Shindo K., Kaji S., Kobayashi S., Takeyama M. (2005). Fabrication of TiAl components by means of hot forging and machining. Intermetallics.

[11] Niu H.Z., Chen Y.Y., Zhang Y.S., Lu J.W., Zhang W., Zhang P.X. (2015). Producing fully-lamellar microstructure for wrought beta-gamma TiAl alloys without single α-phase field. Intermetallics.

[12] Fujitsuna N., Ohyama H., Miyamoto Y., Ashida Y. (1991). Isothermal forging of TiAl-based intermetallic compounds. ISIJ Int..

[13] Erdely P., Staron P., Maawad E., Schell N., Klose J., Mayer S., Clemens H. (2017). Effect of hot rolling and primary annealing on the microstructure and texture of a β-stabilised γ-TiAl based alloy. Acta Mater..

[14] Liu C.T., Wright J.L., Deevi S.C. (2002). Microstructures and properties of a hot-extruded TiAl containing no Cr. Mater. Sci. Eng. A.

[15] Shen G., Furrer D. (2000). Manufacturing of aerospace forgings. J. Mater. Process. Technol..

[16] Zhang Z.X., Qu S.J., Feng A.H., Shen J. (2017). Achieving grain refinement and enhanced mechanical properties in Ti-6Al-4V alloy produced by multidirectional isothermal forging. Mater. Sci. Eng. A.

[17] Zherebtsov S.V., Salishchev G.A., Galeyev R.M., Valiakhmetov O.R., Mironov S.Y., Semiatin S.L. (2004). Production of submicrocrystalline structure in large-scale Ti-6Al-4V billet by warm severe deformation processing. Scripta Mater..

[18] Salishchev G.A., Imayev R.M., Senkov O.N., Imayev V.M., Gabdullin N.K., Shagiev M.R., Kuznetsov A.V., Froes F.H. (2000). Formation of a submicrocrystalline structure in TiAl and Ti_3_Al intermetallics by hot working. Mater. Sci. Eng. A.

[19] Imayev V.M., Salishchev G.A., Shagiev M.R., Kuznetsov A.V., Imayev R.M., Senkov O.N., Froes F.H. (1998). Low-temperature superplasticity of submicrocrystalline Ti-48Al-2Nb-2Cr alloy produced by multiple forging. Scripta Mater..

[20] Sokolovsky V.S., Stepanov N.D., Zherebtsov S.V., Nochovnaya N.A., Panin P.V., Zhilyakova M.A., Popov A.A., Salishchev G.A. (2018). Hot deformation behavior and processing maps of B and Gd containing β-solidified TiAl based alloy. Intermetallics.

[21] Hao Y., Liu J., Li S., Li J., Liu X., Feng X. (2017). Effects of nano-twinning on the deformation and mechanical behaviours of TiAl alloys with distinct microstructure at elevated loading temperatures. Mater. Sci. Eng. A.

[22] Schwaighofer E., Clemens H., Lindemann J., Stark A., Mayer S. (2014). Hot-working behavior of an advanced intermetallic multi-phase γ-TiAl based alloy. Mater. Sci. Eng. A.

[23] Fröbel U., Stark A. (2015). Microstructural Evolution in Gamma Titanium Aluminides During Severe Hot-Working. Metall. Mater. Trans. A.

[24] Niu H.Z., Chen Y.F., Zhang Y.S., Lu J.W., Zhang W., Zhang P.X. (2016). Phase transformation and dynamic recrystallization behavior of a β-solidifying γ-TiAl alloy and its wrought microstructure control. Mater. Des..

[25] Imayev R.M., Salishchev G.A., Senkov O.N., Imayev V.M., Shagiev M.R., Gabdullin N.K., Kuznetsov A.V., Froes F.H. (2001). Low-temperature superplasticity of titanium aluminides. Mater. Sci. Eng. A.

[26] Zhu K., Qu S., Feng A., Sun J., Shen J. (2018). Evolution of the Microstructure and Lamellar Orientation of a β-Solidifying γ-TiAl-Based Alloy during Hot Compression. Metals.

[27] Kainuma R., Fujita Y., Mitsui H., Ohnuma I., Ishida K. (2000). Phase equilibria among α (hcp), β (bcc) and γ (L1_0_) phases in Ti–Al base ternary alloys. Intermetallics.

[28] Zhang W.J., Lorenz U., Appel F. (2000). Recovery, recrystallization and phase transformations during thermomechanical processing and treatment of TiAl-based alloys. Acta Mater..

[29] Sakai T., Belyakov A., Kaibyshev R., Miura H., Jonas J.J. (2014). Dynamic and post-dynamic recrystallization under hot, cold and severe plastic deformation conditions. Prog. Mater Sci..

[30] Appel F. (2012). Phase Transformations and recrystallization processes during synthesis, processing and service of TiAl alloys. Recrystallization.

[31] Liu B., Liu Y., Li Y.P., Zhang W., Chiba A. (2011). Thermomechanical characterization of β-stabilized Ti–45Al–7Nb–0.4W–0.15B alloy. Intermetallics.

[32] Zhang S.Z., Zhang C.J., Du Z.X., Hou Z.P., Lin P., Kong F.T., Chen Y.Y. (2016). Deformation behavior of high Nb containing TiAl based alloy in α + γ two phase field region. Mater. Des..

[33] Zhou H., Kong F., Wang X., Chen Y. (2017). High strength in high Nb containing TiAl alloy sheet with fine duplex microstructure produced by hot pack rolling. J. Alloys Compd..

[34] Blackburn M.J. (1970). Some aspects of phase transformations in titanium alloys. Sci. Technol. Appl. Titan..

[35] Palomares-García A.J., Pérez-Prado M.T., Molina-Aldareguia J.M. (2017). Effect of lamellar orientation on the strength and operating deformation mechanisms of fully lamellar TiAl alloys determined by micropillar compression. Acta Mater..

[36] Imayev R., Imayev V., Oehring M., Appel F. (2005). Microstructural evolution during hot working of Ti aluminide alloys: Influence of phase constitution and initial casting texture. Metall. Mater. Trans. A.

[37] Brockman R.A. (2003). Analysis of elastic-plastic deformation in TiAl polycrystals. Int. J. Plast..

[38] Kishida K., Inui H., Yamaguchi M. (1999). Deformation of PST crystals of a TiAl/Ti_3_Al two-phase alloy at 1000 °C. Intermetallics.

[39] Appel F., Clemens H., Fischer F.D. (2016). Modeling concepts for intermetallic titanium aluminides. Prog. Mater Sci..

[40] Mayer S., Erdely P., Fischer F.D., Holec D., Kastenhuber M., Klein T., Clemens H. (2017). Intermetallic β-Solidifying γ-TiAl Based Alloys—From Fundamental Research to Application: Intermetallic β-Solidifying γ-TiAl Based Alloys. Adv. Eng. Mater..

[41] Bolz S., Oehring M., Lindemann J., Pyczak F., Paul J., Stark A., Lippmann T., Schrüfer S., Roth-Fagaraseanu D., Schreyer A. (2015). Microstructure and mechanical properties of a forged β-solidifying γ TiAl alloy in different heat treatment conditions. Intermetallics.

[42] Fischer F.D., Clemens H., Schaden T., Appel F. (2007). Compressive deformation of lamellar microstructures–a short review. Int. J. Mater. Res..

[43] Yamaguchi M. (1991). Deformation and Recrystallization Behaviour of the TiAl Phase Constituting the TiAl/Ti_3_Al Lamellar Structure of Ti-rich TiAl Compounds. ISIJ Int..

[44] Edwards T.E.J., Di Gioacchino F., Muñoz-Moreno R., Clegg W.J. (2016). Deformation of lamellar TiAl alloys by longitudinal twinning. Scripta Mater..

[45] Huang K., Logé R.E. (2016). A review of dynamic recrystallization phenomena in metallic materials. Mater. Des..

[46] Dumitraschkewitz P., Clemens H., Mayer S., Holec D. (2017). Impact of alloying on stacking fault energies in γ-TiAl. Appl. Sci..

[47] Zhou H., Kong F., Wu K., Wang X., Chen Y. (2017). Hot pack rolling nearly lamellar Ti-44Al-8Nb-(W, B, Y) alloy with different rolling reductions: Lamellar colonies evolution and tensile properties. Mater. Des..

[48] Wan Z., Sun Y., Hu L., Yu H. (2017). Experimental study and numerical simulation of dynamic recrystallization behavior of TiAl-based alloy. Mater. Des..

[49] Fukutomi H., Nomoto A., Osuga Y., Ikeda S., Mecking H. (1996). Analysis of dynamic recrystallization mechanism in γ-TiAl intermetallic compound based on texture measurement. Intermetallics.

